# Thriving despite Parental Physical Abuse in Adolescence: A Two-Wave Latent Transition Analysis on Hedonic and Eudaimonic Violence-Resilience Outcome Indicators

**DOI:** 10.3390/children9040553

**Published:** 2022-04-13

**Authors:** Wassilis Kassis, Dilan Aksoy, Céline Anne Favre, Clarissa Janousch, Sibylle Talmon-Gros Artz

**Affiliations:** 1 Department of Research & Development, School of Education, University of Applied Sciences and Arts Northwestern Switzerland, 5210 Windisch, Switzerland; dilan.aksoy@fhnw.ch (D.A.); celineanne.favre@fhnw.ch (C.A.F.); clarissa.janousch@fhnw.ch (C.J.); 2 School of Child and Youth Care, University of Victoria, Coast Salish Territories, Victoria, BC V8P 5C2, Canada; sartz@uvic.ca

**Keywords:** parental physical abuse, adolescents, violence resilience, hedonic factors, eudaimonic factors

## Abstract

Internationally, about 25% of all children experience physical abuse by their parents. Despite the numerous odds against them, about 30% of adolescents who have experienced even the most serious forms of physical abuse by their parents escape the vicious family violence cycle. In this study, we analyzed longitudinally the data from a sample of *N* = 1767 seventh-grade high school students in Switzerland on physical abuse by their parents. We did this by conducting an online questionnaire twice within the school year. We found that in our sample, about 30% of the participating adolescents’ parents had physically abused them. We considered violence resilience a multi-systemic construct that included the absence of psychopathology on one hand and both forms of well-being (psychological and subjective) on the other. Our latent construct included both feeling good (hedonic indicators, such as high levels of self-esteem and low levels of depression/anxiety and dissociation) and doing well (eudaimonic indicators, such as high levels of self-determination and self-efficacy as well as low levels of aggression toward peers). By applying a person-oriented analytical approach via latent transition analysis with a sub-sample of students who experienced physical abuse (n_w2_ = 523), we identified and compared longitudinally four distinct violence-resilience patterns and their respective trajectories. By applying to the field of resilience, one of the most compelling insights of well-being research (Deci & Ryan, 2001), we identified violence resilience as a complex, multidimensional latent construct that concerns hedonic and eudaimonic well-being and is not solely based on terms of psychopathology.

## 1. Introduction

Research confirms that internationally about 25% of all children experience [1,2,3,4] severe forms of physical abuse by their parents. These numbers seem especially high because they involve significant physical abuse, such as kicks and massive blows, and not only the very common but still very problematic slapping of children on the hand or leg [5]. Studies report a prevalence of 19% in Switzerland [5], 20–25% in the European Union ([5,6,7]), and 28% in the USA [8]. Victimization surveys show that physical victimization of adolescents by parents often goes unreported to the police and young people are less likely than adults to report victimization to the police [9], suggesting that underreporting among young people might be a major policy concern.

Thus far, as the meta-analysis of Stoltenborgh et al. [4] showed, there is no conclusive evidence if the prevalence or incidence of parental abuse is the most appropriate indicator for understanding the respective adolescents’ developmental processes and outcomes. This insight holds for several parental abuse forms, including physical abuse [2,3,4,5,6,7,8,9,10] and sexual abuse [11,12]. Therefore, arguments exist for both incidence and prevalence. Even if the discussion on the severity of parents’ physical abuse forms can be misleading [13], what emerges as important is the difference between slapping on the hand [10] and more force-related parental abuse forms such as kicks, punches, or even strangulations [14]. The importance of including the prevalence of the more severe physical parental abuse forms can be explained by the fact that the burden and the effects of even single episodes of such forms of physical abuse substantially contribute to long-lasting effects on mental health [1,2,4,10].

### 1.1. The Detrimental Effects of Physical Parental Abuse on Adolescents

Parental abuse, also known as child maltreatment, can manifest in many ways, with parental physical abuse being one type that involves the infliction of non-accidental bodily harm. Other types of child maltreatment exist, such as emotional abuse, neglect, and sexual abuse [15]. The effects of the most severe forms of parents’ physical abuse on adolescents’ development are widely described as harming their emotional, personal, and social adjustment and growth, all of which is well documented [16,17,18,19], among which two symptoms, one externalizing (interpersonal aggression toward peers) and one internalizing (depression/anxiety), have been identified as the most central and indicative symptoms in adolescents with experiences of parental physical abuse [1,8,16,20,21]. A growing body of research supports the link between parental physical abuse and the co-occurrence of depression, anxiety [22], and aggression in adolescence [23,24,25,26], and it shows that this co-occurrence can be observed in childhood [25], adolescence, and young adulthood [23,27]. Corroborating data from a meta-analysis of 60 related studies published between 1990 and 2006 also indicate that mental health problems and behavioral problems, such as externalization symptoms in adolescence, are associated with exposure to violence at home [16].

### 1.2. Conceptualizing Violence-Resilience Outcomes of Adolescents with Experience of Parental Physical Abuse

Conceptualizing and evaluating violence-resilience outcomes of adolescents with experience of physical family abuse is a complex endeavor. Bearing in mind that resilience is a relational term and process within and between various systems, not necessarily of equal weights, and that this process involves responsibilities that individuals and social/societal systems share, a more integrative approach is needed. Masten’s [28] suggestion, which we endorse, defines resilience as the ability of a dynamic system, not just of the individual, to adapt successfully to disturbances that jeopardize the system’s function, viability, or development.

Interestingly, in recent years, scholars have widely applied the dual-factor model of mental health to resilience research, which currently focuses specifically on factors contributing to resilience [29,30]. Prior to the dual-factor model [31,32], the conception of mental health included only the absence of psychopathological factors. Nevertheless, from the more recent perspective, psychopathology and subjective well-being are not solely opposite poles of a continuum; rather, they need to be integrated into one common construct, the dual-factor model. However, surprisingly, this has not been the case for violence-resilience outcomes, specifically in identifying and defining violence resilience [33]. This gap in the definition of violence resilience prevents the comparison of research findings across studies, finding insights into the prevalence of violence resilience among maltreated children, and informing prevention and intervention practices and policies to foster violence resilience.

Investigations of the adolescents’ class membership were often implemented in many studies through the inclusion of gender, migration background, and socio-economic status in the analyzed models as socio-demographic predictors. Given that previous findings [34] have shown that being male, having a migration background [35,36], and having a lower socio-economic status can act as risk factors for mental health, their predictive strength for class membership should be identified. Thus far, only limited research exists on the effects of socio-demographic predictors on physically abused adolescents [37]. Additionally, the results on these predictors of adolescents’ resilience-outcome status have been inconclusive [7,38].

Following these thoughts, we believe it is highly important for violence resilience to be conceptualized as adolescent students *feeling good* (the hedonic dimension), doing well, and functioning positively in the school context (the eudaimonic dimension). In this respect, we go one step further than existing resilience research and understand positive adaptation not only as high levels of subjective well-being, academic competence, or the absence of psychopathology. Rather, we assume that violence resilience means both low levels of psychopathology and higher levels of subjective (hedonic) but also psychological (eudaimonic) well-being. At the same time, the person-centered analysis gives space to adolescents who may not be on one side (non-resilient) or the other (resilient) but are somewhere in between; this can be expressed by the dual-factor model. The aim of the paper is therefore to apply the dual-factor model to violence resilience using latent transition analysis, taking into account both hedonic and eudaimonic indicators and controlling for sociodemographic variables.

In general, resilience is the outcome of achieving positive adjustment despite adversity [39], but its presence requires setting clear and agreed upon criteria that describe positive adjustment and outcomes in the face of a specific risk [40], in our case parental physical abuse. Children of parental physical abuse are typically described as resilient, here called violence resilient, and can be identified by specific resilience outcomes, although no single agreed upon resilience definition exists [28]. Identifying violence-resilience outcomes and recognizing adolescents’ resilience after they experience parental physical abuse is a complex endeavor. Even though adolescents experiencing parental abuse commonly show psychopathological symptoms, international research confirms that about one-third of adolescents physically abused by parents do not show psychopathological symptoms, such as depression and aggression toward peers [7,41].

Several studies have focused on the dual-factor model for identifying resilience processes, but fewer have focused on resilience outcomes related to parents’ physical abuse through applying person-centered approaches as latent class (LCA) or latent transition analyses (LTA) for identifying groups based on similar response profiles [42,43,44,45]. The most shared insights included that four classes/profiles on processes toward adolescents’ resilience on mental health could be identified [46,47]: a “flourishing” class with high levels of protective factors and low symptoms, a “vulnerable” group with low protective factors and a middle level of symptoms, a “troubled” group with low protective factors and a high level of symptoms, and “symptomatic but content” group with middle levels of protective factors and high levels of symptoms. In these studies, the “flourishing” and the “troubled” adolescents were most likely to remain in their group while the “symptomatic but content” and the ”vulnerable“ groups were the least stable classes [45,46,47,48].

In many cases, this valuable approach is called “symptomatology-resilience” because it is mainly based on the presence or absence of specific symptoms [49,50,51,52,53]. Despite the bulk of literature on adolescents’ positive development regardless of parental physical abuse, a lively debate continues about defining and measuring violence resilience. Most definitions are based on describing specific psychopathological symptoms rather than their components for a positive life in adolescence, which makes it difficult to draw conclusions, make comparisons, and create broad interventions. Researchers have typically conceptualized resilience following maltreatment in one of three ways: (1) as a personality trait, (2) as outcomes related to adaptive functioning, or (3) as socioecological resources [54]. Even though policymakers and/or academics commonly use the term “violence resilience” in connection with adolescents who have experienced parental physical abuse, it is still inconsistently defined. Additionally, because of the dynamic development children undergo during adolescence and the particular changes in their violence-resilience status, resilience sustainability over time is of the utmost importance.

### 1.3. Applying Learnings from Well-Being Research to Identify Adolescents’ Violence-Resilience Components: A New Model Combining Well-Being and Resilience Research

Identifying the internalizing and externalizing symptoms as resilience indicators among adolescents who have experienced parental physical abuse is driven by the evidence-based insight that these very symptoms hinder beneficial development [21,37] and, therefore, hinder the forming of a positive life. In their review of resilience after maltreatment, Yule et al. [55], who conducted a meta-analysis of 118 studies on protective factors involving 101,592 participants, noted that in resilience research, positive adaptation is understood as, among other things, the absence or low presence of psychopathology, the achievement of competencies in important domains such as school, and high levels of subjective well-being. In this study, we consider the insight that the absence of negative outcomes, such as depression and aggression, at time 1 (t1) is not an adequate evidence of adolescents’ positive development despite having experienced parental physical abuse and that this resilience state at t2 will be the same.

We see this assumption as a theoretical shortcoming that perpetuates an important beta error, a false negative, and has us retain the null hypothesis when it is actually wrong. Meaning, under these conditions, adolescents are identified incorrectly as violence resilient even if they are not, and we submit that we cannot assume that just because adolescents lack internalizing and externalizing symptoms at one point in time, they are doing well. Therefore, we hypothesize that our beta error-based conclusions and falsely accepted violence-resilience status led to misspecifications of the ongoing need for fostering processes among these adolescents. While rightly criticizing an exclusively psychopathology-oriented view on resilience, too often the mistake is made of underestimating that adolescents’ psychopathology in general [56] or for specific social groups [57] (including components of optimal experience and functioning) is indeed a valid and needed point, but as stated here, it is not the only element in identifying adolescent students’ violence-resilience outcomes.

To help apply a non-exclusively psychopathology-oriented approach, we adapt one of the most compelling insights of well-being research [58,59] to the field of resilience and identify violence resilience as a complex and multidimensional latent construct that includes feeling good and doing well. Consequently, we suggest that adolescent students’ violence-resilience indicators should refer to their present and future lives because these factors relate to their emotional, social, and academic performance, and therefore, they entail more than just general satisfaction with one’s life or positive performance in certain areas. 

Interestingly, these two core dimensions of well-being (hedonic and eudaimonic) have been operationalized in very different ways. In their review of the research on experienced well-being, Martela and Sheldon [60] identified at least 63 eudaimonic constructs and regarded the satisfaction of psychological needs as the common core connecting the hedonic and eudaimonic dimensions. Therefore, we expect hedonic and eudaimonic aspects of violence resilience will correlate as distinct aspects of resilience.

Deciding on resilience criteria can be very difficult because it can involve numerous indicators. Following Luthar et al. [39], we argue that for adolescents to be resilient, they must excel in multiple adjustment domains. Considering there is no such thing as general resilience but only resilience related to a specific developmental burden [61], these specific resilience-outcome indicators of optimal experience and functioning for adolescent students who have experienced physical abuse by their parents must be explicitly geared to this very specific developmental burden. Therefore, we have to seek domain-specific violence-resilience indicators despite parental physical abuse among early adolescent students and conceptualize the respective hedonic and eudaimonic aspects accordingly. Additionally, as resilience is not only domain specific as related to the content (the particular burden), resilience processes can only be addressed and fostered appropriately via topological specificity. Because of this, we need to keep in mind that for a child and youth care worker dealing with families, a eudaimonic aspect such as “functioning well” in a family is not the same as it is for a school social worker who is aiming to support “functioning well” at school even if the two fields (family and school) are related.

Feeling good, meaning the presence of positive and the absence of negative affect, represents the hedonic aspects of violence resilience among adolescent students, emphasizes the strive for positive experiences, and consists of cognitive and affective components, such as higher levels of self-esteem [62] and lower levels of depression/anxiety [3] and dissociation [63]. The association between self-esteem and violence resilience in adolescence is well documented and has been reported as an assessment of an individual’s global worthiness and one of the most decisive determinants of violence resilience in adolescence [3]. High school students with higher levels of self-esteem feel that school challenges threaten them less [64] because they evaluate their own personality via salient attributes, thereby ensuring a positive representation of themselves and asserting their global dignity. Therefore, self-esteem works in adolescence both as an outcome and as a buffer throughout challenging times [65], which is especially important for adolescents who have experienced parental physical abuse. Adolescent students’ violence resilience is perceived as not only an experience of pleasant emotions at school but also the absence of or rather low levels of negative affect. Here, “negative affect” refers to addressing negative emotions, such as sadness and fear [66]. Abuse experiences jeopardize the optimal development of affect regulation skills; therefore, it may become more challenging for troubled adolescents to regulate and differentiate affective experiences [15]. Furthermore, parental physical abuse contributes to the development of internalizing symptoms. A large body of research demonstrates that youth with experiences of abuse show increased levels of internalizing symptoms, such as high levels of depression [7,67,68,69], anxiety [70,71,72], and dissociation [63,73,74,75]; therefore, low levels of depression, anxiety, and dissociation are a central emotional hedonic component of violence resilience.

However, adolescent students’ violence resilience goes beyond the experience of positive and the absence of negative affect. It also involves a eudaimonic element, which includes promoting positive social skills in early adolescence and positive functioning in their school settings as central to environmental mastery [76]. This understanding of resilience includes three dimensions: mastery at school, indicated by high self-efficacy; fulfilling basic psychological needs; and lack of or low levels of aggression toward peers.

Mastery at school, the first eudaimonic dimension, focuses on perceived self-efficacy [77,78] as a generalized concept of behavioral expectations and is based on being able to handle the demands and challenges that students face in school settings. Andretta and McKay [79] showed that self-efficacy was a key variable in well-being processes. Higher levels of perceived self-efficacy are favorable for setting and achieving goals [47] and support both motivation and very concrete activities at school [80]. Consequently, students with higher levels of self-efficacy tend not just to set higher goals but are also both more efficient and more realistic in planning their actions at school. This is key for adolescents who have experienced parental physical abuse because this kind of mastery helps them regain control over their lives. Jerusalem and Schwarzer [81] referenced general perceived self-efficacy as a core indicator of the ability to cope with life challenges in adolescence, and by extension, with detrimental experiences of familial violence.

The second dimension, fulfilling basic psychological needs, includes positive relationships with others, autonomy, and growth in academic competence as essential parts of positive school performance [82]. Prominently, Deci and Ryan [83] identified autonomy, experiences of competence, and social relatedness as basic psychological needs. The need for competence focuses on reliable instrumentalities leading to specific outcomes, the need for autonomy focuses on students’ aspirations to experience the self as the origin of their actions at school, and the need for social relatedness encompasses the universal urge to experience interrelatedness and feel securely connected at school. Fulfilling these basic psychological needs is vital for adolescents who have experienced parental physical abuse because of their very crucial need for effective functioning and psychological health [84].

The third eudaimonic dimension of violence resilience in the context of well-being is low aggression toward peers. Children and adolescents who have experienced parental violence consider aggression an appropriate response and more often make snap judgments about hostile intentions, and exhibit more aggressive responses compared to those who have not had these experiences [85], which in turn may lead to a higher risk of peers re-victimizing them [86]. Because peer aggression is a highly important consequence of experiences with parental violence, low levels of peer aggression importantly indicate resilient development from a violence-resilience perspective [1,8,16,20,21] because victimization and aggression are both negatively associated with well-being [87].

### 1.4. Present Study: Violence-Resilience Stability and Change over Time 

As noted above, resilience sustainability over time must be better understood and considered regarding this phenomenon’s definitions, which makes establishing the stability of violence-resilience pathways over time desirable [88]. The stability of violence-resilience outcomes regarding hedonic and eudaimonic aspects is entirely unknown. We propose that we need to ask what happens after the first “ordinary magic”, as Masten [40] describes resilience, is detected at wave 1 among adolescent students with whom we have established the two hedonic and eudaimonic aspects on various extents, and with that, examine how resilience and the corresponding pathway look in wave 2 for adolescents with experiences of parental physical abuse. We need to ask if wave 2 simply shows a continuation of resilience patterns already experienced at wave 1, or if that depends on the different patterns of hedonic and eudaimonic aspects.

The questions that we investigate here address that longitudinal studies on the pathways of resilient adolescents with experiences of parental physical abuse are internationally rare [83,89,90,91,92].

Consequently, the development of violence resilience throughout adolescence remains unclear. To address this issue, we examined longitudinally the combined contribution of eudaimonic and hedonic factors in predicting violence-resilience patterns in early adolescence and identified their respective trajectories. Using latent class and latent transition analysis [42,93] as well as person-oriented procedures, we expected to estimate and understand adolescent students’ continuity of violence-resilience levels at two time points, specifically whether the transition occurs developmentally forward (e.g., transition to higher resilience levels) or backward (e.g., transition to lower resilience levels or remain at the same level). This methodology allows grouping subjects into distinct classes based on the violence-resilience indicators included in the analysis and then estimating the probability that a particular subject (also a person-oriented method) is a member of that class.

Although identifying adolescent violence-resilience patterns at a given time is an important first step, knowing whether, why, and how these patterns change longitudinally over time is essential in designing possible school-specific prevention and intervention programs. Therefore, understanding the interplay between the introduced hedonic and eudaimonic indicators and the potential changes over time is necessary. Research suggests that low socio-economic status, migration background, and female gender predict violence resilience. Therefore, we assume these factors might influence the membership in different groups that show each pattern of violence resilience and its stability and change after one year at school.

Thus far, we have almost no knowledge convincingly showing how nonpathological violence-resilience outcomes despite parental physical abuse in early adolescence will develop over time and, in particular, on how these patterns change longitudinally over time for different adolescent groups. Thus, we conducted this study to fill these gaps in knowledge through discovering violence-resilience outcome patterns over time.

Because of the study’s exploratory character and because the introduced conceptualization of hedonic and eudaimonic indicators for identifying resilience outcomes of adolescents whose parents physically abused them has not been applied thus far, we investigated four exploratory hypotheses. First, we predicted that the introduced three hedonic and three eudaimonic indicators would allow identifying distinct resilience-outcome classes of adolescents whose parents physically abused them. Based on previous findings, we expected to find four resilience-outcome classes as the optimal number of groups for both time points. Second, following already existing research on mental health [68], we expected to identify a resilient, a non-resilient, a vulnerable, and a symptomatic but content class. Third, considering resilience is a state and not a trait, we expect fluctuations between the to-be-identified resilience-outcome classes at different time points. We expect the resilient and the non-resilient adolescents will most likely remain in their class, while the symptomatic but content and the troubled will be the least stable classes [45,68]. Fourth, we expect socio-demographic predictors, such as gender, migration background, and socio-economic status, will influence the participating adolescents’ class membership in the model.

## 2. Materials and Methods

### 2.1. Study and Sample

The analyzed data come from a two-waves longitudinal sample (which two survey waves within the next two years will follow) of a broader study on adolescents’ violence-resilience pathways despite experiencing parental physical abuse, which was conducted in the early autumn of 2020 (*M_age_wave* 1 = 11.76 (*SD_age_wave* 1 = 0.64)) and early summer 2021 (*M_age_wave* 1 = 12.28 (*SD_age_wave* 1 = 0.56)) with representative convenient samples. Schools were contacted to recruit full classes of seventh-grade high school students from German-speaking Switzerland. Consent forms were obtained from students and their caregivers. No incentives were given. The research ethics committee at the University in Zurich, Switzerland authorized the project. On the day of the study, the research team members gave a short oral introduction about the online survey to the students who were present in the participating 142 classes in 44 high schools and the students completed the questionnaire in about 60 min. For the analysis stage, we drew “abuse” sub-samples of both waves (wave 1 *n* = 560; wave 2 *n* = 523), consisting of adolescents who reported having experienced parental physical abuse at least once in their lifetime.

We ran *t*-tests (see Table 1) to analyze for mean differences on socio-demographic variables and the six applied measures between the two waves, including overall samples for wave 1 (N = 1858) and wave 2 (N = 1764), and as for the specific sub-samples of adolescents having experienced physical parental abuse, we used the sub-samples “abuse”, (Wave 1_*n* = 560, Wave 2_*n* = 523). Referring first to the three introduced socio-demographic variables, overall, we identified only small effects (all displayed Cohen’s d are far lower than <0.5) between the overall samples and the respective “abuse” sub-samples for both waves. Even when considering this, we detected significantly higher percentages of adolescents with a migration background and a lower socio- economic level in the “abuse” sub-samples compared to those in the overall samples for both waves.

When comparing the levels of the six indicators of the overall samples and the corresponding “abuse” sub-samples, we identified, for both waves, very similar outcomes. Concerning the hedonic indicators, the overall samples for both waves displayed higher self-esteem and lower levels of depression/anxiety and dissociation than in the respective “abuse” sub-samples. The eudaimonic indicators reproduced a similar picture. There were higher levels of self-efficacy and self-determination and lower levels of aggression toward peers for both overall samples in comparison to the “abuse” sub-samples for both waves. 

The attrition of the “abuse” sub-samples from wave 1 (*n* = 560) to wave 2 (*n* = 523) of only 6.61% is very low. Between wave 1 and wave 2 participants, no significant differences existed regarding the tested socio-demographic variables to (gender_*t*(560) = 0.904, *p* > 0.05; migration background *t*(560) = −1.483, *p* > 0.05; socio-economic status *t*(560) = −0.859, *p* > 0.05). Due to this, we consider the two samples comparable to the participating students.

### 2.2. Measures

#### 2.2.1. Prevalence of Parental Family Physical Abuse

The single-item indicator on the prevalence of parental physical abuse indicates that adolescents reported having experienced parental physical abuse at least once in their lifetime. Response categories for prevalence of parental physical abuse were dichotomized as no (0) or yes (1).

#### 2.2.2. The Six Latent Class/Latent Transition Indicators

##### The Three Hedonic Indicators

*Self-esteem* was assessed according to the Rosenberg Self-Esteem Scale [94] for assessing an individual’s global worthiness evaluation. This tool is comprised of a five-item short scale, with higher scores indicating higher self-esteem. The items were rated on a four-point Likert scale ranging from 1 = “not at all” to 4 = “extremely” (Cα_wave 1 = 0.90; Cα_wave 2 = 0.92). Respondents were asked to rate questions such as, “In total, I am confident in myself.” For the LCA/LTA we performed a median split (MED_wave 1 = 3.00; MED_wave 2 = 3.00) and dichotomized this as either (0) lower levels or (1) higher levels of self-esteem. 

*Symptoms of anxiety and depression* were assessed through 24 items that were part of the Hopkins Symptom Checklist [95] (e.g., “I feel fear” and “Thoughts of ending my life”). From the original 25-item scale version, one item (“Loss of sexual interest or pleasure”) was not included because of the participants’ young age of approximately 12–14 years. The items were rated on a four-point Likert scale ranging from 1 = “not at all” to 4 = “extremely,” (Cα_wave 1 = 0.96; Cα_wave 2 = 0.96). For the LCA/LTA, we performed a median split (MED_wave 1 = 1.62; MED_wave 2 = 1.65) and dichotomized this as either (0) lower levels or (1) higher levels of symptoms of anxiety and depression. 

*Dissociation*: The items for assessing dissociation as a disruption or discontinuity of consciousness were measured on a four-item short scale (Dissociation Tension Scale Acute) [96]. This scale consisted of one item each of depersonalization, somatoform, derealization, and analgesia. Participants could rate these items on a four-point Likert scale ranging from 1 = “not at all” to 4 = “very much” (Cα_wave 1 = 0.80; Cα_wave 2 = 0.85). For the LCA/LTA, we performed a median split (MED_wave 1 = 1.00; MED_wave 2 = 1.00) and dichotomized this as either (0) lower levels or (1) higher levels of symptoms of dissociation.

##### The Three Eudaimonic Indicators

Self-efficacy: The General Self-Efficacy Scale is a psychometric scale that Schwarzer and Jerusalem [81] developed. It is designed to assess optimistic self-belief regarding coping with various challenging demands in life (e.g., “I am confident that I could deal efficiently with unexpected events”). The six-item short scale (Cα_wave 1 = 0.88; Cα_wave 2 = 0.90) was measured on a four-point Likert scale (range: 1 = not true to 4 = completely true). For the LCA/LTA, we performed a median split (MED_wave 1 = 2.83; MED_wave 2 = 2.83) and dichotomized this as either (0) lower levels or (1) higher levels of self-efficacy.

Self-determination: Following Deci and Ryan’s [82] self-determination theory (SDT) on basic human psychological needs, we measured the three subscales of autonomy, competence, and relatedness on short scales with three items each (e.g., on the subscale autonomy, “I was free to do things in my own way”). The nine-item scale (α_wave 1 = 0.87; α_wave 2 = 0.90) was measured on a four-point Likert scale (range: 1 = not true at all to 4 = completely true). For the LCA/LTA, we performed a median split (MED_wave 1 = 3.00; MED_wave 2 = 3.11), and dichotomized this as either (0) lower levels or (1) higher levels of self-determination.

Aggression toward peers: To assess overt (e.g., threatening to hit classmates or physically hurt them in other ways) and covert aggression (e.g., spreading harmful rumors about classmates) toward peers in the classroom as perpetrators, we applied the German Self-Report Behaviour Aggression-Opposition Scale [97], which consists of nine items. We measured it on a four-point Likert scale: 1 = “never happened,” 2 = “once or twice per month,” 3 = “once per week,” and 4 = “more than once per week” since the school year started (α_wave 1 = 0.83; α_wave 2 = 0.84). For the LCA/LTA, we performed a median split (MED_wave 1 = 1.2; MED_wave 2 = 1.44), and dichotomized this as either (0) lower levels or (1) higher levels of aggression toward peers.

##### The Three Covariates

Gender: Students’ gender was assessed with three response options (0 = *boys*, 1 = *girls*, and 3 = *other*). As only three students out of 1987 chose “other” we worked without these three cases.

Socio-economic status (SES): Students’ SES was used as a proxy for students’ socioeconomic background and was merged as a mean score using four indicators (1 lowest to 3 highest SES, Cα = 0.71). Information on parental education was gathered from the two questions: “What is the highest level of school education that your mother has completed?” and “What is the highest level of school education that your father has completed?” (ranging from 1 = *Primary School/ Junior High School*, 2 = *Vocational Education/General High-school Certificate* to 3 = *University Degree/Higher Education*). Additionally, we incorporated the information on the books the adolescents (ranging from 1 = 0–5 books, 2 = 6–30 books to 3 = 31 books on) and family (ranging from 1 = 0–10 books, 2 = 11–100 books to 3 = 101 books on) owned. 

Migration background (MB): Not having a migration background meant the student was born in Switzerland and they possessed only the Swiss passport. Having a migration background was operationalized such that one or more of the aforementioned conditions did not apply (0 no MB, 1 with MB).

### 2.3. Analytic Strategy

This study’s aim was three-fold. Firstly, we tested the introduced violence-resilience outcomes conceptualization through using both hedonic and eudaimonic aspects. Secondly, we identified adolescent violence-resilience outcome patterns and knowing how these patterns change over time as an essential step for designing prevention and intervention programs. Thirdly, we tested if the new categorization applied via LCA/LTA reduced the beta error of incorrectly identifying adolescents as violence resilient even if they are not.

To empirically classify the six introduced latent variables (three to hedonic and three to eudaimonic domains) to violence-resilience subgroups based on observations that appeared to be similarly related to hedonic and eudaimonic aspects, we applied LCA and LTA as typological person-oriented approaches [42,93,98]. Unlike variable-centered analyses, LCA/LTA allow for identifying specific persons’ latent profiles [48]. Both LCA and LTA include categorical indicators to identify different groups in empirical data [93]. Through an iterative process of choosing the optimal number of profiles between a one-profile solution to six-profile solutions, we determined the optimal solution. We assigned the individuals to the different patterns based on their posterior probabilities for class membership and tested these through the classification stability of the respective violence-resilience patterns of the specific individuals for wave 1 and wave 2.

Missing data were estimated using the full information maximum likelihood method. LCA/LTA analyses were conducted with maximum likelihood estimation, and due to non-normal distributions, with robust standard errors [99]. To avoid local solutions, we increased for all LCAs and LTAs performed the random starts to 1000 and final optimizations to 100 [48].

We conducted consecutive LCA/LTA series to identify the definite number of profiles. We applied different criteria for the model selection. First, the entropy value indicated the certainty in the estimatiFFon with values above 0.7 considered sufficient [100,101,102,103]. Second, for the information criteria, we used criterion such as the Akaike information criterion (AIC), Bayesian (Schwarz) information criterion (BIC)**,** and Sample-Adjusted BIC (SABIC), with the smaller values fitting the model better [93,102]. For the LCA, we additionally applied model fit criteria as the Vuong-Lo-Mendell-Rubin Likelihood Ration test (LMR-LRT), the Lo-Mendell-Rubin Adjusted Likelihood Ratio test (aLMR-LRT) [104], and the Bootstrapped Likelihood Ratio test (BLRT) with significant *p*-values indicating an improvement compared with the previous model with k-1 classes [102]. However, we chose the final model for an LCA/LPA based on a mixture of statistical indicators, extant theoretical considerations, and the rule of deference to more constrained and parsimonious models [102].

Therefore, we conducted this study’s statistical analysis in four steps. First, wave 1 versus wave 2 survey differences in the six applied measures (self-esteem, depression/anxiety, dissociation, self-efficacy, self-determination, and aggression toward peers) were examined using paired samples *t*-tests. Second, we identified students’ resilience outcome classes through computing separately for wave 1 and for wave 2 LCA using the six classification variables. Additionally, we applied an invariance analysis across time to ensure the reliability for the identified number of resilience outcomes (configural invariance) as well as the same relevance of the resilience-outcome patterns (metric invariance) for both study waves. Third, we ran LTA to indicate significant differences in the longitudinal classification variables on the identified resilience-outcome patterns. Fourth, we included the covariates gender, migration background, and socio-economic level to multinomial logistic regression analyses to predict the identified latent status membership. For the *t*-tests, we used SPSS (Version 24; IBM Corp., New York, USA, 2016), all other analyses conducted were assessed using *M*plus version 8.6 [105].

## 3. Results

### 3.1. Analytic Step One: Differences of All Measures between the Two Waves

We ran *t*-tests for paired samples (see Table 2) to analyze for mean differences between the two waves of the six applied measures for our sub-sample (wave 1_*n* = 560, wave 2_*n* = 523). Overall, moderate effects for depression/anxiety, dissociation, and aggression toward peers for all three measures were at significantly higher levels at wave 2, but no effects on the other three measures were displayed.

### 3.2. Analytic Step Two: Identifying Resilience-Outcome Classes via LCA for Both Waves

For each of the two waves, we tested for resilience-outcome patterns via computing two separate LCAs. We applied the introduced six classification variables, three on hedonic and three on eudaimonic aspects, to determine via LCA the optimal number of classes for each wave sharing the same pattern of resilience outcome for each detected class. Based on their response similarity in the measured three hedonic and three eudaimonic indicators for each wave (wave 1_*n* = 560; wave 1_*n* = 523) separately. LCAs for both waves were conducted for a range of two to six latent classes to determine significantly differing resilience-outcome classes for adolescents experiencing parental physical abuse.

When LCA was applied, for the non-nested models and choosing for the models’ selection goodness of fit, the sample-adjusted Bayesian information criterion (aBIC) with a lower value indicated a more appropriate fit, and entropy indicated the estimation’s accuracy, with models having sufficient values above 0.7 [93,102]. The final LCA model decision was based on a mixture of statistical indicators, theoretical considerations, and the rule of deference to more parsimonious models [101,106].

**Figure 1:**
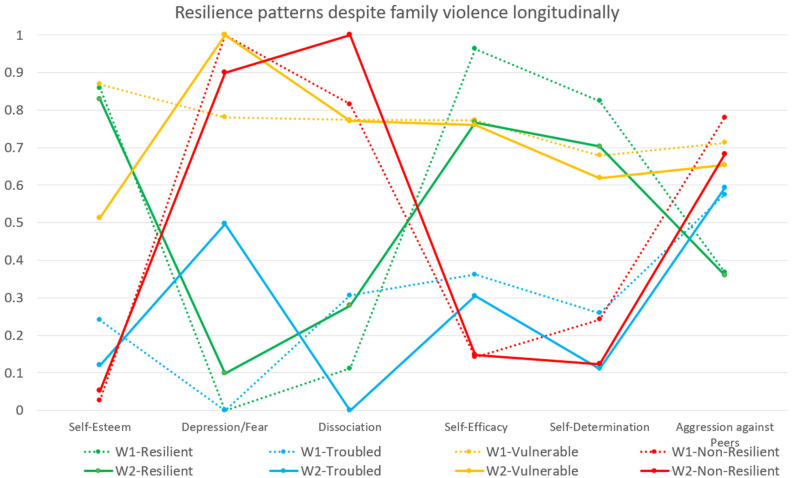
Item response probabilities and violence resilience-outcome patterns for both waves.

Based on the three hedonic indicators (self-esteem, depression/anxiety, and dissociation) and three eudaimonic indicators (self-efficacy, self-determination, and aggression toward peers) we applied a series of LCAs for both waves to group students into empirically distinct resilience-outcome classes for adolescents having experienced parental physical abuse. When parallel analyzing the data for both waves, the aBIC scores dropped between the three and four class solutions for both waves and the still-significant tests (VLMR, aLMR, and the bootstrap likelihood ratio test (BLRT) indicated an improvement, supporting a four over three class solution. Between classes four and five, there was an aBIC rise (wave 1_ΔBIC = 12; wave 2_ΔBIC = 12). For wave 1, the two performed tests (VLMR, aLMR) indicated no improvement between the class four to class five solution, only the BLRT test was significant. For wave 2, all three performed tests (VLMR, aLMR, BLRT) indicated no improvement between the class four to class five solution. Therefore, a four class solution was selected for both waves (see Figure 1). 

**Figure 2:**
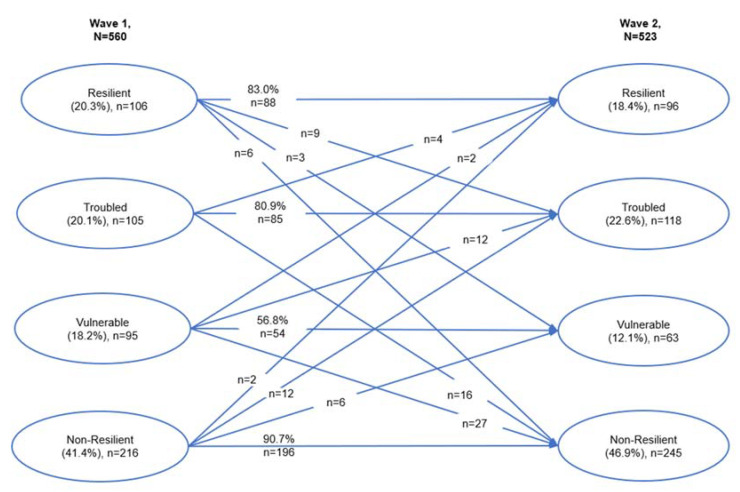
Classes transition over time.

Regarding the distribution of all six indicators on the four identified classes for both waves (see Figure 2), we identified vast similarities between the two waves. We detected a class called “resilient” (wave 1 = 20.3%; wave 2 = 18.4%), a class called “troubled” (wave 1 = 20.1%; wave 2 = 22.6%), a class called “vulnerable” (wave 1 = 18.2%; wave 2 = 12.1%), and a class called “non-resilient” (wave 1 = 41.4%; wave 2 = 46.9%) resilience-outcome classes for both waves. The indicators’ probabilities (see Figure 2) on the respective levels were highly comparable on the three hedonic and three eudaimonic indicators, supporting the chosen classes solution for both waves.

For both waves, we noticed the students’ immense resilience outcome differences on hedonic and eudaimonic indicators when comparing the resilience classes (hedonic indicators: high levels of self-esteem and low levels of depression/anxiety and dissociation; and eudaimonic indicators: high levels of both, self-efficacy as for self-determination, and low levels of aggression toward peers) to the non-resilience classes (hedonic indicators: low levels of self-esteem and high levels of depression/anxiety and dissociation; and eudaimonic indicators: low levels of both, self-efficacy as for self-determination, and high levels of aggression toward peers). 

In addition, for both waves, we detected a class called “vulnerable” (hedonic indicators: middle levels of self-esteem and high levels of depression/anxiety and dissociation; and higher levels of all three eudaimonic indicators: self-efficacy, self-determination, and aggression toward peers). We called the fourth detected class for both waves “troubled”, which had a very distinctive profile on the proliferation of the introduced hedonic indicators (low levels of self-esteem, middle to low levels of depression/anxiety, and low levels of dissociation) and eudaimonic indicators (low levels of both, self-efficacy as for self-determination, and high levels of aggression toward peers). Quite deliberately, we do not call this group “aggressive,” even though the students had higher levels of aggression toward peers, because they also had very low levels of self-esteem (hedonic indicator) and self-efficacy and self-determination (both eudaimonic indicators).

Based on the identified four resilience outcome patterns for both waves, we tested for measurement invariance [64] across time in the number of resilience outcome patterns (configural invariance) that could be analyzed for wave 1 and wave 2. We also tested whether the loadings on the respective latent classes were invariant, thus ensuring that the factors’ structures, that is, the four patterns, were the same for both waves (metric invariance). When testing for metric measurement invariance, we identified a nonsignificant chi-square difference test (Δchi^2^ [24] = 35.71, *p* > 0.05.), thereby establishing the same relevance for the four resilience outcome patterns for both waves. Ensuring metric invariance was the first approach necessary to compare the four resilience outcome patterns over the school year.

To summarize the invariance testing results, we found the same number of resilience outcome patterns and hedonic and eudaimonic dimensions present for adolescents having experienced parental physical abuse across both waves over a one-year period. In terms of content, this indicates the four introduced and empirically analyzed violence-resilience outcome patterns provided an empirically reliable measure on two waves longitudinally.

Having established this structure similarity for both waves, we could then approach the third analysis step on testing stability and change among different patterns of well-being via applying LTA.

### 3.3. Analytic Step Three: LTA to Indicate Significant Differences in the Longitudinal Classification Variables on the Identified Resilience-Outcome Patterns

In step three, we ran an LTA to indicate significant differences in the longitudinal classification variables on the identified patterns. LTA, the longitudinal extension of LCA, is a statistical tool that fulfills the needs of modeling adolescents’ violence-resilience outcome transitions over time [93,102]. After determining separately that the optimal number of classes at each time point was four (see analysis step two), we performed an LTA to estimate the probabilities of violence-resilience outcome pattern changes over time from one latent class to another [93]. This process can estimate the continuity of resilience outcomes at adjacent time points. At this statistical step, change is represented via the probability of transitioning to a latent violence-resilience outcome status at wave 2, given latent status membership at wave 1. In addition, it explores whether the same latent status can be identified in both wave 1 and wave 2 [42,101]. 

We ran an LTA using the previously mentioned three hedonic and three eudaimonic classification variables (for model fits see Table 3). The LTA was conducted for a range of two to six latent classes to test if the conditional response probabilities had been constrained to be time invariant.

The aBIC dropped between the three and four class solution (−Δ55) and the corresponding aBIC stability (−Δ0) from the four to the five class solution indicated a four class solution as the appropriate one. The detected samples for the respective solutions (see Table 4) supported this with the five class and six class solutions having numerous sub-samples with far too few (<*n* = 50) allocated students to the particular sub-samples. Due to the sub-sample sizes and the rule of deference to more constrained models, a four class solution was selected for the longitudinal analyzes via LTA. 

Regarding the distribution of the four classes for both waves (see Table 5), we identified only very low changes over time for the “resilient” class (of −1.9% from wave 1 to wave 2) and the “vulnerable” class (of 2.5% from wave 1 to wave 2). We noticed moderate changes, particularly a decrease in the “vulnerable” class of −6.1% from wave 1 to wave 2, and an increase in the “non-resilient” class of 5.5% from wave 1 to wave 2.

Regarding comparing the classes’ stability over one school year, a multilayered picture can be identified (see Figure 2). Concerning the stability over time, three (“resilient,” “troubled,” and “non-resilient”) out of four classes showed a remarkable immobility of higher than 80% of the students being reassigned to the same class. In contrast, only 56.8% of the students being assigned at wave 1 to the “vulnerable” class were at the same class at wave 2.

Interestingly, when looking closer at these changes over time from the “vulnerable” class, only a negligible number (*n* = 2, as 2.1%) moved to the “resilient” class, 12.6% (*n* = 12) were assigned to the “troubled” class, and almost every third student (*n* = 27, as 28.9%) transitioned to the “non-resilient” class. In terms of “ordinary magic”, as Masten [40] described resilience, regarding only an almost negligible proportion of the participating students, less than 2% (*n* = 8), transitioned to the “resilient” class: 3.8% (*n* = 4) of the “troubled”, 2.1% (*n* = 2) of the “vulnerable”, and just about 0.9% (*n* = 2) of the “non-resilient” adolescents.

### 3.4. Analytic Step Four: Covariates Gender, Migration Background, and Socio-Economic Level Were Included to Multinomial Logistic Regression Analyses to Predict the Identified Latent Status Membership

After identifying the classes for both waves, we applied a multinomial logistic regression. Our analysis included, for both waves, socio-demographical covariates that could plausibly relate to resilience-outcome pattern variations despite experiencing parental physical abuse (see Table 6). Gender, migration background, and SES were included as socio-demographic predictors to the identified latent status membership.

The socio-demographic variables showed for both waves (see Table 6) a very low prediction to the identified LCA patterns. Notably, only gender, but neither migration background nor SES, showed any prediction to the identified resilience-outcome patterns. For both waves, a highly significant number of females was assigned to the resilient group compared to the non-resilient group. For wave 1, compared to the non-resilient group, a highly significant number of females compared to males were in the vulnerable group. Compared to the non-resilient group for both waves, significantly more females than males were in the vulnerable group. Just for wave 2, compared to the resilient group, significantly more males than females were assigned to the vulnerable group. Likewise, just for wave 2, compared to the resilient group, significantly more males than females were assigned to the vulnerable group.

## 4. Discussion

Internationally, about 25% of the adolescents experience physical abuse by their parents, who are their primary caregivers [4]. Exposure to physically abusive parents creates conditions in which maladaptive development in adolescents is highly likely. However, studies consistently report that, contrary to expectations, a proportion of these adolescents are neither showing externalizing nor internalizing behaviors, suggesting that they may be considered “resilient” according to the generally accepted definition. Increasingly, researchers are questioning whether this symptom-focus is perhaps too general, and we too questioned the negative symptoms-oriented understanding of violence-resilience outcomes. Based on this, we asked the following question: How adaptive are youths with experiences of violence when positive outcomes are considered alongside negative outcomes? To answer this question, we drew on findings from well-being research and used not only dimensions of subjective well-being but also psychological well-being as indicators of violence resilience [59]. We identified violence-resilience as a complex and multidimensional latent construct that encompasses feeling good and doing well: In doing so, we used three hedonic and three eudaimonic indicators to identify distinct violence-resilience outcome classes of youth whose parents physically abuse them. Considering that general resilience does not exist, we had to understand first the domain-specific content of violence-resilience. Following theoretical considerations, we introduced self-esteem, depression/anxiety, and dissociation as three hedonic indicators for feeling good despite having experienced parental physical abuse. Additionally, we applied the three eudaimonic indicators self-efficacy, self-determination, and aggression toward peers for positive functioning in adolescence and environmental mastery. 

Following the study’s exploratory character and the introduced conceptualization of hedonic and eudaimonic indicators for identifying distinct violence-resilience outcomes, we applied a longitudinal analysis via LCAs/LTA. Additionally, considering resilience is not a trait but a fluctuating state at different time points [107], we ran longitudinally analyses for these resilience outcomes. To achieve this goal, we applied LTA as a person-centered approach for identifying homogenous groups based on similar resilience-outcome response patterns [93] despite parental physical abuse. 

For both waves, we identified a prevalence of about 30% of the most severe forms of parental physical abuse, showing a higher proliferation than the expected prevalence of about 20–25% [3,5]. We assumed this to be the often-discussed COVID-19 effect with families and their members being under higher individual, social, and financial strains [108,109,110]. The identified higher levels of depression/anxiety, dissociation, and peer aggression at wave 2 support this conclusion.

Through applying a two-wave longitudinal design and analyzing the data of both waves via LCAs, we detected distinct resilience-outcome patterns following the introduced theoretical line of reasoning and similar studies on the dual-factor model of mental health [111]. We did not only identify a resilient (high levels of feeling good and doing well) and a non-resilient group (low levels of feeling good and doing well) as well as replicated the results of mental health studies [46], but we also identified two very interesting, and thus far, not discussed violence-resilience outcome groups.

First, a class we called “troubled” (see related results on mental health as introduced by Xiong et al. [47]) was made up of adolescents with a very mixed profile on hedonic (low self-esteem, middle to low depression/anxiety, and low dissociation levels) and eudaimonic (low levels of self-efficacy and self-determination but high levels of aggression toward peers) indicators. This group (about every fifth adolescent in our sample was being physically abused), when viewed through the new introduced theoretical consideration, is not only specifically aggressive but is also characterized by very low levels of the hedonic indicators and the two additional eudaimonic indicators self-efficacy and self-determination. These adolescents were “running” very scarce on any additional hedonic and eudaimonic resources, and because of that, they also had very low probabilities on changing to higher resilience-outcome levels. This assumption was empirically validated through this group’s high stability over time and additionally through the result that almost every sixth adolescent of this group transitioned by wave 2 to the non-resilient group.

The second newly introduced group for violence-resilience outcomes is the group we called “vulnerable,” replicating Kelly et al.’s [46] and Xiong et al.’s [47] studies, which named this group symptomatic but content. The group is characterized by very heterogeneous profiles of both the hedonic (high levels of self-esteem, as with depression/anxiety and dissociation) and the eudaimonic (high levels of self-efficacy, as with self-determination and aggression toward peers) indicators. This group’s adolescents displayed a highly symptomatic profile but initially, and at least just on the data surface, seemed content with this situation. As Diamantopoulou et al. [112] was able to identify, an exaggerated self-esteem can be related to aggressive behavior in adolescence. This phenomenon on the self-esteem paradox has later been described as “defensive egotism” [113] and refers to a compensation model of aggression in adolescence being driven by a defensive personality. Thanks to our two wave longitudinal design and the applied LTA, we identified this group’s enormous instability (just about every second adolescent of this group did transition to another class) toward a lower resilience-outcome level, indicating that a characterization as “content” as Kelly et al. [46] and Xiong et al. [47] originally assigned despite the high levels of depression/anxiety, dissociation, and aggression toward peers would be thoroughly unfitting. We highlight the finding on this group, that from wave 1 to wave 2, about every tenth student moved to the “troubled” group and more than every fourth moved to the non-resilient group. 

Referring to the stability of the state of the adolescents over time, through our analyses, we confirmed the expected stability of the resilient (83%) and the non-resilient (90.7%) adolescents, while the vulnerable and the troubled classes were the least stable [45,111]. Returning to the resilient group (high levels of feeling good and of doing well), we detected a very high stability over time, indicating an anchored resilience state over the analyzed school year. The highest stability of the identified resilience-outcome groups was detected for the non-resilient adolescents with almost no adolescents moving to other resilience-outcome classes, especially to the resilient group. The “ordinary magic of resilience,” as Masten [40,114] describes it, did not seem to apply for them. Referring to violence-resilience in adolescence when having experienced parental physical abuse, any notion of “ordinary magic”-resilience does not seem to exist, thus it almost does not happen, and we suggest that it must be implemented and fostered pointedly.

Connected to the focus on thriving not only surviving parental physical abuse, we incorporated at least two main issues often detected in violence-resilience research. First, the chosen person-centered methods are whole-person approaches and through including further indicators, we supported the aim to understand the specific adolescents’ violence-resilience outcomes latent reality behind the symptomatology that manifests on the surface. Through undertaking this and incorporating a general sample, not a clinical sample, we avoided stating “the kids are all right” because they are not experiencing higher levels of internalizing or externalizing effects despite having experienced such massive physical abuses by their primary caregivers (this could also be related to the actual world-political situation when stating that the absence of war is not a sufficient indicator of freedom). Secondly, we established this latent person-oriented approach of hedonic and eudaimonic indicators on violence-resilience as a state not a trait and gained insights on adolescents’ fluctuating resilience thriving not only surviving parental physical abuse over time, which is a desideratum [115].

Our results have shown conclusively and for two waves within a school year in early adolescence that neither depression/anxiety, or as it has been mainly called “internalizing symptoms,” nor aggression against peers as “externalizing symptoms” could be called empirically sufficient or even content-wise adequate predictors for violence-resilience despite parental physical abuse. 

The new categorization that developed from well-being research provided us the opportunity to identify the hedonic and the eudaimonic indicators. Along with the original indicator, the respective three indicators displayed an enormous variation. This can be seen especially, but certainly not exclusively, in the composition of the “troubled” and “vulnerable” groups, over the four identified resilience-outcome patterns. 

Because previous findings have shown that gender, migration background, and SES can act as risk factors, they might be highly influential on resilience-outcome classes [34]. For both waves, the socio-demographic variables showed a very low prediction of the specific LCA class membership. Notably, only gender (and not migration-background nor SES) showed any prediction of the identified resilience-outcome patterns. In particular for both waves, significantly more females were assigned to the resilient group compared to the non-resilient group. Additionally, and again for both waves, compared to the non-resilient group, significantly more females than males were in the vulnerable group. These results are only partly consistent with previous studies showing these covariates’ effects [116]. Other international studies identifying SES and familial wealth as not being predictors of parental physical abuse [1,3,7] corroborate our results. We still have to take into consideration that testing gender as binary, as we did, still results in an enormous reduction of the existing gender variations and requires an intersectional approach [117].

It would be both difficult and dangerous to load the violence-resilience’s outcome burden on the adolescents’ shoulders, even when applying a whole-person approach via the chosen hedonic and eudaimonic indicators. We must acknowledge that the best way to support adolescents’ lives in the first place is not to hurt them emotionally, physically, or sexually. The proliferation of massive parental physical abuse tells a different story.

## 5. Limitations

Dichotomizing data for LCA/LTA always restricts findings. Through applying a median split, participants are divided into two groups, and through that, the standard deviation is reduced artificially [118,119], but a mandatory step to conduct LCAs and LTA implemented. At the same time, in our study, by applying these person-centered methods and developing profiles within individuals, we minimized classification dichotomies as common when using variable-centered methods [120].

The results of the two-wave longitudinal analyses that we performed (gathering data twice within the first high school year) will have to be verified by the following two data waves (waves 3 and 4), each of which will be gathered at the end of grades 8 and 9.

The study focused on how resilience outcomes appeared, but we did not analyze processual factors leading to the four identified resilience outcomes. Because of our data, we only applied a two-waves longitudinal design, and from that, we could not analyze these processes. 

In our study, we worked with the physical abuse prevalence rate that the adolescents reported. It is of course a limitation not having additional data from parents or social services, but as Stoltenborgh et al. [4] showed, the prevalence rates from informant studies were lower compared to self-report studies. Additionally, self-report studies of adolescents are considered very accurate [1].

By focusing on parental physical abuse prevalence and not on experienced incidence, we could not consider additional physical abuse characteristics as frequency and duration [13]. One of the most compelling methodological problems on this issue is that incidence reports in early adolescence, even if they appear to be abuse reports that are more accurate at first glance, have to be focused on very recent events. Following Stoltenborgh’s [4] and Brown’s [109] insights, we assumed that the forms of parental physical abuse that are more severe were of higher importance. For early adolescence, and when having at least three wave longitudinal data on parental physical abuse, it would be desirable to understand the specific contributions of prevalence and incidence data on a more nuanced understanding of the long-lasting effects on adolescents’ development [1,4,109].

We dichotomized migration background by information on the countries of origin and birth. Such a formal categorization, which is not a self-identification of the adolescents [121], comes with a loss of information because migrants are very diverse in terms of their migration generation, legal migrant status depending on the very specific laws of the country, and the status of their countries of origin being possibly connected to prejudices and social strains [122].

## Figures and Tables

**Table 1:** Wave 1 and wave 2 sample mean levels (and standard deviations) of socio-demographic variables and all observed variables for the LCAs/LTA between the overall samples and the sub-samples of adolescences having experienced physical parental abuse.

		Wave 1			Wave 2		
		Overall Sample N = 1858	Sub-Sample «Abuse» *n* = 560	Cohen’s *d*	Overall Sample N = 1764	Sub-Sample «Abuse» *n* = 523	Cohen’s *d*
Variables	Range	*M* (*SD*)	*M* (*SD*)		*M* (*SD*)	*M* (*SD*)	
Gender	1–2 (1 male; 2 female)	1.50 (0.50)	1.56 * (0.50)	−0.11	1.50 (0.50)	1.53 (0.50)	-
	% female	51.3%	55.9%		51.3%	52.6%	
Migration Background	0–1 (0 no MB, 1 with MB)	0.32 (0.47)	0.44 *** (0.50)	−0.25	0.30 (0.46)	0.44 *** (0.50)	−0.30
	% with MB	35.7%	43.8%		33.0%	44.4%	
Socio-Economic Status	1–3 (1 lowest to 3 highest)	2.11 (0.55)	2.00 *** (0.56)	0.20	2.13 (0.57)	1.98 *** (0.59)	0.25
	% lowest level	21.0%	25.9%		23.7%	28.2%	
	% middle level	60.8%	59.2%		58.8%	58.0%	
	% highest level	18.2%	14.8%		17.6%	13.8%	
Self-Esteem	1–4 ^1^	3.08 (0.72)	2.85 *** (0.{Citation})	0.31	3.12 (0.75)	2.80 *** (0.81)	0.43
Depression/Anxiety	1–4 ^1^	1.73 (0.60)	1.99 *** (0.69)	−0.42	1.73 (0.66)	2.10 *** (0.77)	−0.54
Dissociation	1–4 ^1^	1.32 (0.55)	1.55 *** (0.70)	−0.39	1.32 (0.59)	1.63 *** (0.79)	−0.47
Self-Efficacy	1–4 ^1^	2.82 (0.62)	2.71 ** (0.66)	0.17	2.84 (0.69)	2.68 *** (0.69)	0.24
Self-Determination	1–4 ^1^	3.07 (0.61)	2.92 *** (0.64)	0.23	3.10 (0.66)	2.89 *** (0.64)	0.32
Aggression Against Peers	1–4 ^1^	1.31 (0.39)	1.51 *** (0.50)	−0.46	1.35 (0.39)	1.62 *** (0.57)	−0.59

^1^ higher numbers indicate higher levels of the respective indicator; * *p* < 0.05; ** *p* < 0.01; *** *p* < 0.001 between the wave’s overall sample and sub-sample.

**Table 2:** Paired *t*-tests, wave 1 (*n* = 523) and wave 2 (*n* = 560) sub-sample mean levels (and standard deviations) of all six latent class/latent transition indicators.

Indicators	Range	Wave 1 *M (SD)*	Wave 2 *M (SD)*	Cohen’s *d*
Self-Esteem	1–4	2.84 (0.80)	2.82 (0.80)	-
Depression/Anxiety	1–4	1.96 (0.67)	2.11 (0.77) ***	0.234
Dissociation	1–4	1.53 (0.68)	1.63 (0.79) **	0.145
Self-Efficacy	1–4	2.73 (0.67)	2.69 (0.71)	-
Self-Determination	1–4	2.94 (0.64)	2.89 (0.69)	-
Aggression Against Peers	1–4	1.49 (0.50)	1.62 (0.57) ***	0.197

** *p* < 0.01; *** *p* < 0.001 between wave 1 and wave 2.

**Table 3:** Latent class analysis model fit statistics to select the number of classes of resilience at school for both waves sequentially.

Wave 1								Wave 2						
Classes	AIC (dF)	aBIC	VLMR	aLMR	BLRT	Entropy	Samples	AIC (dF)	aBIC	VLMR	aLMR	BLRT	Entropy	Samples
2	3565 (13)	3580	<0.001	<0.001	<0.001	0.61	241/313	3431 (13)	3445	<0.001	<0.001	<0.001	0.61	187/333
3	3501 (20)	3524	<0.01	<0.01	<0.001	0.71	160/299/95	3398 (20)	3419	<0.05	<0.05	<0.001	0.62	249/169/102
4	3481 (27)	3511	<0.05	<0.05	>0.001	0.74	99/112/68/275	3383 (27)	3413	<0.01	<0.01	<0.001	0.70	91/120/86/223
5	3484 (34)	3523	>0.05	>0.05	>0.05	0.73	43/80/71/87/273	3389 (34)	3425	>0.05	>0.05	>0.05	0.68	16/88/93/236/87
6	3487 (41)	3534	<0.05	<0.05	>0.05	0.81	73/34/31/274/61/81	3398 (41)	3443	>0.05	>0.05	>0.05	0.72	26/8/243/74/76/93

AIC = Akaike information criterion; aBIC = sample-size adjusted Bayesian information criterion; VLMR = Vuong–Lo–Mendell–Rubin Likelihood Ratio Test; aLMR = Lo–Mendell–Rubin Adjusted LRT Test.

**Table 4:** Latent transition analysis model fit statistics to select longitudinally the number of classes of resilience at school.

Classes	AIC (df)	aBIC	Entropy	Samples
2	6645 (15)	6661	0.80	c1: 339/183; c2: 350/172
3	6500 (26)	6529	0.77	c1: 90/268/164; c2: 58/307/157
4	6432 (39)	6474	0.71	c1: 106/105/95/216; c2: 96/118/63/245
5	6415 (54)	6474	0.78	c1: 93/223/43/69/94; c2: 54/241/67/68/92

AIC = Akaike information criterion; aBIC = adjusted Bayesian information criterion.

**Table 5:** Estimated longitudinal probabilities of the four resilience patterns by latent transition analysis.

Resilience Pattern	Wave 1	Wave 2	ΔW2-W1
Resilient	20.3%	18.4%	−1.9%
Troubled	20.1%	22.6%	+2.5%
Vulnerable	18.2%	12.1%	−6.1%
Non-Resilient	41.4%	46.9%	+5.5%

**Table 6:** Wave 1 and wave 2, multinomial logistic regression of socio-demographic covariates to the identified latent status membership on the four resilience-outcome-patterns.

		Socio-Demographic Factors			
		Wave 1		Wave 2	
Resilience-Outcome-Patterns		B (SE)	OR	B (SE)	OR
Reference Pattern «non-resilient» vs. Pattern «resilient»	Intercept	−4.27 (1.04) ***	-	−3.48 (0.85)	-
	Gender (1 male; 2 female)	1.30 *** (0.39)	3.69	1.93 *** (0.34)	6.91
	Migration Background (0 no MB, 1 with MB)	−0.35 (0.43)	-	−0.40 (0.32)	-
	Socio-Economic Status (1 lowest to 3 highest)	0.55 (0.32)	-	0.29 (0.26)	-
Reference Pattern «non-resilient» vs. Pattern «vulnerable»	Intercept	−3.89 *** (0.88)		−1.07 (0.93)	
	Gender (1 male; 2 female)	1.21 *** (0.32)	3.34	0.65 (0.41)	-
	Migration Background (0 no MB, 1 with MB)	0.38 (0.32)	-	−0.36 (0.48)	-
	Socio-Economic Status (1 lowest to 3 highest)	0.58 (0.32)	-	−0.22 (0.40)	-
Reference Pattern «non-resilient» vs. Pattern «troubled»	Intercept	−3.38 *** (0.86)		−2.01 * (0.79)	
	Gender (1 male; 2 female)	1.49 *** (0.33)	4.46	0.87 ** (0.31)	2.39
	Migration Background (0 no MB, 1 with MB)	−0.09 (0.33)	-	−0.33 (0.32)	-
	Socio-Economic Status (1 lowest to 3 highest)	0.08 (0.29)	-	0.05 (0.27)	-
Reference Pattern «resilient» vs. Pattern «vulnerable»	Intercept	0.39 (1.28)		2.40 * (0.97)	
	Gender (1 male; 2 female)	−0.09 (0.47)	-	−1.28 ** (0.44)	0.28
	Migration Background (0 no MB, 1 with MB)	0.74 (0.51)	-	0.04 (0.46)	-
	Socio-Economic Status (1 lowest to 3 highest)	0.03 (0.40)	-	−0.25 (0.37)	-
Reference Pattern «resilient» vs. Pattern «troubled»	Intercept	0.89 (1.38)		1.47 (1.01)	
	Gender (1 male; 2 female)	0.19 (0.51)	-	−1.06 * (0.42)	0.35
	Migration Background (0 no MB, 1 with MB)	0.27 (0.56)	-	0.07 (0.38)	-
	Socio-Economic Status (1 lowest to 3 highest)	−0.47 (0.43)	-	0.02 (0.33)	-
Reference Pattern «vulnerable» vs. Pattern «troubled»	Intercept	0.51 (1.08)		0.93 (0.98)	
	Gender (1 male; 2 female)	0.29 (0.41)	-	0.22 (0.44)	-
	Migration Background (0 no MB, 1 with MB)	−0.47 (0.39)	-	0.02 (0.49)	-
	Socio-Economic Status (1 lowest to 3 highest)	−0.50 (0.36)	-	0.27 (0.42)	-

Estimate = *β* from R3STEP analysis; *** *p* < 0.001; ** *p* < 0.01; * *p* < 0.05.; OR only displayed when the corresponding comparisons are significant.

## Data Availability

The raw data supporting the conclusions of this article will be made available by the authors, without undue reservation, upon completion of the project in 2023.
